# Pleiotropic Effects of Bacterial Small Alarmone Synthetases: Underscoring the Dual-Domain Small Alarmone Synthetases in *Mycobacterium smegmatis*

**DOI:** 10.3389/fmicb.2020.594024

**Published:** 2020-10-14

**Authors:** Sushma Krishnan, Dipankar Chatterji

**Affiliations:** Molecular Biophysics Unit, Indian Institute of Science, Bangalore, India

**Keywords:** short alarmone, (p)ppGpp, pGpp, stress response, R-loop, replication stress, ssRNA, RNase HII

## Abstract

The nucleotide alarmone (p)ppGpp, signaling the stringent response, is known for more than 5 decades. The cellular turnover of the alarmone is regulated by RelA/SpoT homolog (RSH) superfamily of enzymes. There are long RSHs (RelA, SpoT, and Rel) and short RSHs [small alarmone synthetases (SAS) and small alarmone hydrolases (SAH)]. Long RSHs are multidomain proteins with (p)ppGpp synthesis, hydrolysis, and regulatory functions. Short RSHs are single-domain proteins with a single (p)ppGpp synthesis/hydrolysis function with few exceptions having two domains. Mycobacterial RelZ is a dual-domain SAS with RNase HII and the (p)ppGpp synthetase activity. SAS is known to impact multiple cellular functions independently and in accordance with the long RSH. Few SAS in bacteria including RelZ synthesize pGpp, the third small alarmone, along with the conventional (p)ppGpp. SAS can act as an RNA-binding protein for the negative allosteric inhibition of (p)ppGpp synthesis. Here, we initially recap the important features and molecular functions of different SAS that are previously characterized to understand the obligation for the “alarmone pool” produced by the long and short RSHs. Then, we focus on the RelZ, especially the combined functions of RNase HII and (p)ppGpp synthesis from a single polypeptide to connect with the recent findings of SAS as an RNA-binding protein. Finally, we conclude with the possibilities of using single-stranded RNA (ssRNA) as an additional therapeutic strategy to combat the persistent infections by inhibiting the redundant (p)ppGpp synthetases.

## Introduction

In 1969, Cashel and Gallant first identified the nucleotide alarmone molecules, guanosine-5', 3'-pentaphosphate (pppGpp) and guanosine-5', 3' -tetraphosphate (ppGpp), from amino acid-starved *Escherichia coli* (Cashel and Gallant, 1969). Intracellular levels of (p)ppGpp are controlled by RelA/SpoT homolog (RSH) proteins as a response to various external and internal stresses encountered by the organisms (Chatterji and Ojha, 2001; Potrykus and Cashel, 2008; Srivatsan and Wang, 2008; Wu and Xie, 2009; Roghanian et al., 2019). This is a direct pathway of stringent response in which the (p)ppGpp signals the massive switch from energy-consuming to energy-conserving process (Potrykus and Cashel, 2008; Abranches et al., 2009; Kriel et al., 2012; Gaca et al., 2013; Hauryliuk et al., 2015; Liu et al., 2015; Steinchen and Bange, 2016). In Gram-negative organisms, beta and gamma subgroups of proteobacteria carry two such enzymes where the main role of RelA is (p)ppGpp synthesis and SpoT in hydrolysis. SpoT can also synthesize (p)ppGpp and is therefore bifunctional (Xiao et al., 1991; Gentry and Cashel, 1996). In Gram-positive organisms, there is a single bifunctional Rel enzyme which synthesizes and degrades (p)ppGpp (Mittenhuber, 2001; Jain et al., 2006; Srivatsan and Wang 2008; Takada et al., 2020).

Apart from these classical, long, multidomain RSHs, few small RSH homologs were identified in organisms ranging from bacteria to plants. They are mostly monodomain, monofunctional proteins either with short alarmone synthetase (SAS) or short alarmone hydrolase (SAH) activity (Sun et al., 2010; Atkinson et al., 2011; Jimmy et al., 2020). The discovery of SAS and SAH opened a new line of research, to understand the indirect pathways of stress response induced by cues such as cell wall antibiotics, acid, alkali, hydrogen peroxide, ethanol, etc. (Horsburgh and Moir, 1999; Cao et al., 2002; Mascher et al., 2003; Thackray and Moir, 2003; Weinrick et al., 2004; D’Elia et al., 2009; Kim et al., 2012; Geiger et al., 2014; Pando et al., 2017). We have identified a dual-domain SAS in *Mycobacterium smegmatis* with RNase HII and (p)ppGpp synthesis activity (Murdeshwar and Chatterji, 2012).

Small alarmone synthetases play an important role to maintain the basal level of (p)ppGpp, which in turn induces the virulence of the pathogenic bacteria. The “(p)ppGpp pool” produced by the long and short RSH enzymes (Ronneau and Hallez, 2019) and the consecutive guanosine triphosphate (GTP) depletion are the key factors determining the formation of bacterial persister cells (Fung et al., 2020). Therefore, understanding the SAS-mediated synthesis and regulation of (p)ppGpp is the need of the hour to modify the current antibacterial therapy.

## Salient Features of Small Alarmone Synthetases

Small alarmone synthetases were identified in bacteria, such as *Streptococcus mutans*, *Bacillus subtilis*, *Enterococcus faecalis*, *Streptococcus pneumoniae*, *Mycobacterium smegmatis*, *Staphylococcus aureus*, *Corynebacterium glutamicum*, *Clostridium difficile*, *Vibrio cholerae*, and *Pseudomonas aeruginosa*. There are two highly homologous SAS proteins in bacteria and are named as RelP (SAS2, YwaC) and RelQ (SAS1, YjbM). Jimmy et al. (2020) reported the recent classification of SAS and identified 30 subfamilies. The functions of five of these subgroup enzymes were experimentally validated (Table 1) and found to be present in toxin–antitoxin (TA) system (Jimmy et al., 2020). The list of previously characterized bacterial SAS is given in Table 1. Their domain structures are given in Figure 1.

**Table 1:** Short alarmone synthetases in bacteria.

Name of the bacteria	SAS type	Function	References
Gram-positive bacteria			
*Streptococcus mutans*	RelP	Stronger (p)ppGpp synthetic activity than RelQ Induced by H_2_O_2_ stress	Lemos et al., 2004, 2007, 2008; Seaton et al., 2011; Kim et al., 2012
	RelQ	Involved in acid and oxidative stresses	
*Bacillus subtilis*	RelP	Induced by alkaline stress Dimerization of 70S ribosome	Nanamiya et al., 2008; Natori et al., 2009; Tagami et al., 2012; Schafer et al., 2020
	RelQ	Synthesize pGpp Contribute to thermoresistant phenotype	
*Enterococcus faecalis*	RelQ	Vancomycin tolerance Virulence Synthesize pGpp Negative allosteric regulation by ssRNA	Abranches et al., 2009; Gaca et al., 2013, 2015a,b; Beljantseva et al., 2017; Colomer-Winter et al., 2018
*Streptococcus pneumoniae*	RelP	Both are low active (p)ppGpp synthetase	Battesti and Bouveret, 2009; Kazmierczak et al., 2009
	RelQ		
*Mycobacterium smegmatis*	RelZ	Bifunctional protein with (p)ppGpp synthetase and RNase HII activity Induced under replication stress Synthesize pGpp Regulation by ssRNA	Murdeshwar and Chatterji, 2012; Krishnan et al., 2016; Petchiappan et al., 2020
*Staphylococcus aureus*	RelP	Cell envelope stress Synthesize pGpp	Geiger et al., 2014; Gratani et al., 2018; Manav et al., 2018; Bhawini et al., 2019; Yang et al., 2019; Li et al., 2020
	RelQ	Mediates β-lactum resistance in methicillin-resistant strains Synthesize pGpp	
*Corynebacterium glutamicum*	RelP_Cg_	Role in primary nucleotide metabolism Respond to low temperatures	Ruwe et al., 2017
	RelS_Cg_	Synthesize pGpp	
*Clostridium difficile*	RelQ	Antibiotic resistance	Pokhrel et al., 2020
*Bacillus subtilis*	PhRel2	All of these are grouped as toxSASs since they are toxic component of TA system	Jimmy et al., 2020; Dedrick et al., 2017
*Coprobacillus* sp.,	FaRel2		
*Mycobacterium* phage Phrann	PhRel	PhRel helps in preventing the superinfection by other bacteriophages	
*Cellulomonas marina*	FaRel		
*Mycobacterium tuberculosis*	CapRel		
Gram-negative bacteria			
*Vibrio cholerae*	RelV	Regulate basal level of (p)ppGpp Induced upon glucose or fatty acid starvation	Das and Bhadra, 2008; Das et al., 2009; Dasgupta et al., 2014
*Pseudomonas aeruginosa*	Tas1 (RelV)	Important role in interbacterial antagonism	Ahmad et al., 2019

**Figure 1:** Domain structure of long RelA/SpoT homolog (RSH) and short alarmone synthetase (SAS). SAS proteins have only (p)ppGpp synthetic domain (~25–29 kDa), the hydrolysis and regulatory domains are absent. RelS is a 39.8-kDa protein with extended synthetase domain than other SAS. RelZ is a 64.5-kDa protein with RNase HII domain. The hydrolysis and regulatory domains are TGS, ThrRs, GTPase, and SpoT; H, helical domain; ZFD, zinc finger domain; CC, conserved cysteine; RRM, ribosome recognition motif; ACT, aspartokinase, chorismate mutase, and TyrA. Tas1 synthetase is a toxin effector domain, and proline-alanine-alanine-arginine (PAAR) is a toxin delivery domain; PhRel, FaRel, PhRel2, FaRel2, and CapRel are known as ToxSAS because of their presence in toxin–antitoxin (TA) module.

## Molecular Functions of Small Alarmone Synthetases

**Figure 2:**
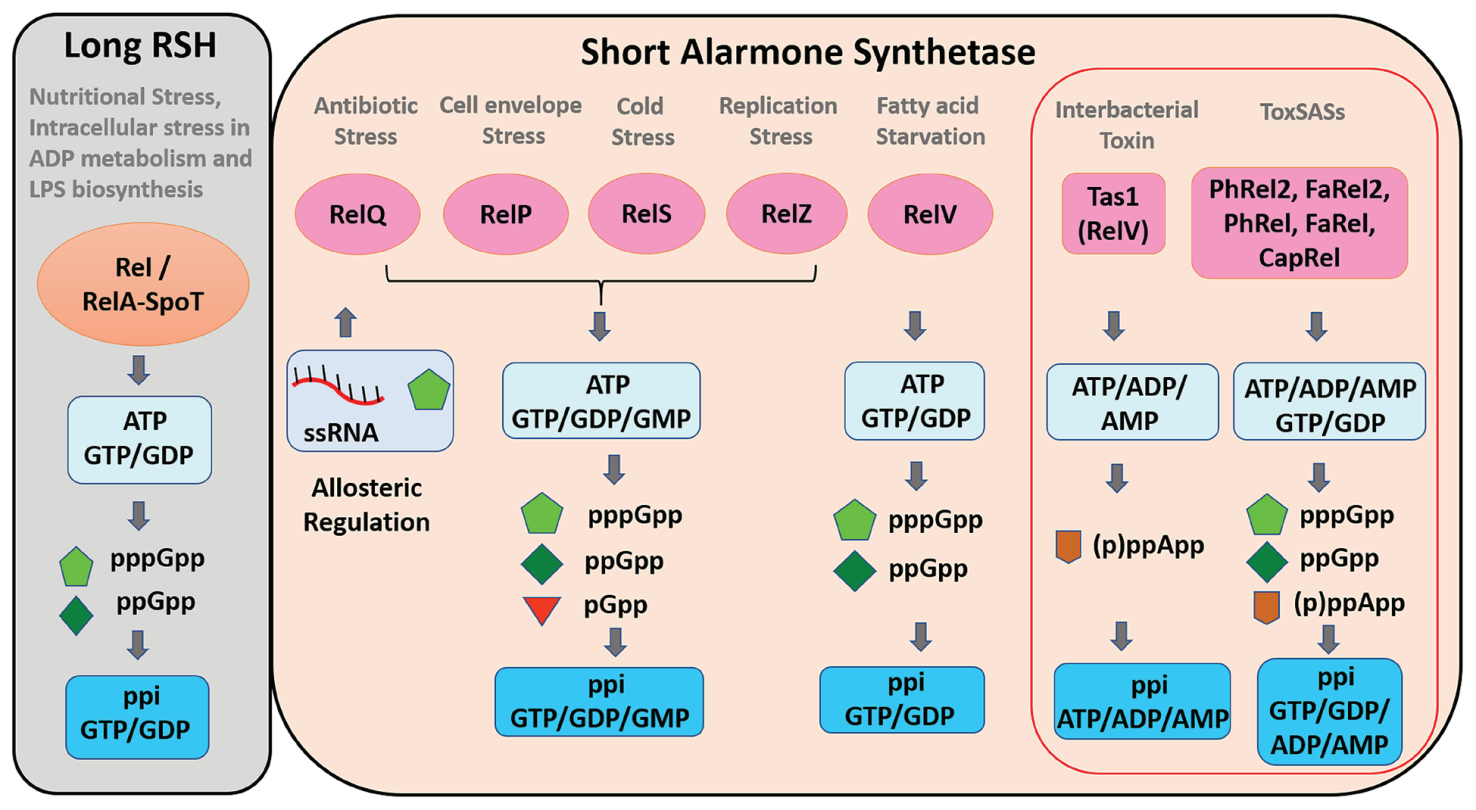
Functions of long RSH and short alarmone synthetase. Long RSH, Rel/RelA-SpoT, synthesize pppGpp and ppGpp using GTP/GDP as substrates during nutritional stress. SAS protein expression is induced by various stress signals. SAS Rels synthesize pGpp in addition to pppGpp and ppGpp using GTP/GDP/GMP as substrates. pppGpp and ssRNA bind to RelQ and mediate the allosteric regulation. The pppGpp synthesis activity of RelZ is also inhibited by RNA and pppGpp. Tas1 synthesize (p)ppApp using ATP/ADP/AMP as substrates. ToxSASs (not Tas1) synthesize both (p)ppGpp and (p)ppApp. ToxSASs are represented within red outlined box.

Different SAS have different roles because they are induced by different signals (Figure 2). RSH is activated mostly under starvation and to the intracellular imbalances involving LPS biosynthesis and ADP metabolism (Roghanian et al., 2019), whereas SAS may respond to various types of environmental stimuli (Figure 2). Maintaining the basal level of (p)ppGpp is important for protection against different kinds of stresses, especially antibiotics stress. Most of the SAS proteins prefer guanosine diphosphate (GDP) to GTP as a substrate (Murdeshwar and Chatterji, 2012; Geiger et al., 2014; Gaca et al., 2015b). Rel and SAS are involved in the allosteric regulation of guanosine and GTP biosynthesis (Gaca et al., 2013; Bittner et al., 2014; Kriel et al., 2014).

## RelP and RelQ

RelP and RelQ share nearly 50% sequence similarity at the amino acid level. *relP/relQ* genes are upregulated due to various stress cues, such as cell envelope (Cao et al., 2002; D’Elia et al., 2009; Geiger et al., 2014), alkali (Nanamiya et al., 2008), ethanol (Pando et al., 2017), high salt, acidic, heat, and hydrogen peroxide (Thackray and Moir, 2003; Weinrick et al., 2004; Kim et al., 2012; Zweers et al., 2012). The first SAS proteins (RelP and RelQ) were identified in *S. mutans* (Lemos et al., 2004, 2007). During oxidative and acidic stress, RelP helped to slow the growth of the bacteria (Kim et al., 2012). Rel inactivation did not yield a lethal phenotype of *S. mutans*, and the basal level of (p)ppGpp was not increased through RelP and RelQ-dependent (p)ppGpp synthesis (Lemos et al., 2007). This could be due to the existence of an alternative mechanism for (p)ppGpp degradation in Streptococci (Lemos et al., 2007). In *B. subtilis*, RelP and RelQ have growth phase-dependent regulation. *relQ* is mainly transcribed in mid-exponential phase and it slows down its expression in the late-exponential phase; in addition, the *relP* is highly induced at this phase (Nanamiya et al., 2008). The (p)ppGpp synthesis of *B. subtilis* RelP is induced by alkaline stress (Nanamiya et al., 2008). In *E. faecalis*, only RelQ synthesizes (p)ppGpp apart from Rel (Abranches et al., 2009). The ∆*relAQ* strain showed significant sensitivity to vancomycin, ampicillin, and norfloxacin (Abranches et al., 2009; Gaca et al., 2013). In *E. faecalis*, (p)ppGpp-mediated antibiotic resistance happens at a concentration below the required value to mount stringent response. *Staphylococcus aureus* contains RelP and RelQ homologs. The expression of these two SAS is induced upon cell wall stress with vancomycin and ampicillin. The presence of three (p)ppGpp synthetases plays a significant role in the development of methicillin-resistant *S. aureus* (MRSA). Like the RelP of *S. mutans*, the RelP of *S. aureus* is also a more potent (p)ppGpp synthetase (Geiger et al., 2014). *Clostridium difficile* has a RelQ that is induced by antibiotic stress. There is a 2-fold upregulation of *relQ* after exposure to ampicillin and clindamycin, which explains the role of RelQ in antibiotic survival (Pokhrel et al., 2020).

Crystal structures of RelP and RelQ from *B. subtilis* and *S. aureus* revealed the homotetramer structures with highly similar monomers and homologs of (p)ppGpp synthetase domains. RelQ activity is inhibited by ssRNA (Beljantseva et al., 2017) and positively regulated by pppGpp (not ppGpp), whereas RelP is not allosterically regulated by (p)ppGpp. This is because of the difference in the conformation of the substrate binding site of these proteins. The RelQ, homotetramer of *B. subtilis*, has a distinct cleft in its center for the binding of two allosteric (p)ppGpp molecules (Steinchen et al., 2015, 2018; Steinchen and Bange, 2016). RelP has been shown to influence the formation of ribosome dimers to inactivate the translation of metabolic pathway (Tagami et al., 2012).

## RelS

*Corynebacterium glutamicum* has two SAS proteins (Figure 2), represented as RelP_Cg_ and RelS_Cg_ (actRel subgroup). The SAS protein encoded by cg2324 is named as RelS and shares sequence similarity with the (p)ppGpp synthetase domain of RelQ. (p)ppGpp synthesis activity is not found for RelP_Cg_. The maximum activity of the RelS_Cg_ is obtained at a temperature below optimum; therefore, it is assumed that (p)ppGpp is synthesized in response to low temperatures (Ruwe et al., 2017).

## ToxSASs

Many SAS subfamilies were identified in conserved bicistronic operon of TA system from *actinobacteria*, firmicutes, and *proteobacteria*. Five of these SAS were demonstrated to be the toxic component of the TA system and hence named as toxSASs (Jimmy et al., 2020). They are *B. subtilis* PhRel2, *Coprobacillus* sp., FaRel2, *Mycobacterium* phage Phrann PhRel, *Cellulomonas marina* FaRel, and *Mycobacterium tuberculosis* CapRel (Figures 1, 2). The toxicity of the toxSASs was neutralized by the six adjacent antitoxin proteins, among which five are specific to corresponding toxSASs and *C. marina* FaRel2 can neutralize all the five toxSASs. The specific function of the toxSASs is not identified, except of PhRel (also known as Gp29), which plays a role in preventing the superinfection by other bacteriophages (Dedrick et al., 2017).

## RelV

RelV (relA-like (p)ppGpp synthetase domain coding gene in vibrios) shared poor homology with RelP and RelQ, because the bacteria itself are phylogenetically different from firmicutes, but there is a high conservation of amino acid residues in the synthetase domains of RelV, RelP, and RelQ. In *V. cholerae*, RelV can produce (p)ppGpp upon glucose or fatty acid starvation (Das and Bhadra, 2008; Das et al., 2009; Dasgupta et al., 2014). Another RelV subfamily homolog Tas1 was identified in *P. aeruginosa*. Tas1 RSH domain is encoded within a large conserved T6SS cluster (type 6 secretion system) and fused to a toxin delivery domain (Figure 2), which exhibits its toxic effect on another competitor cell, thus playing an important function in interbacterial antagonism (Ahmad et al., 2019).

## RelZ (MS_RHII-RSD)

**Figure 3:**
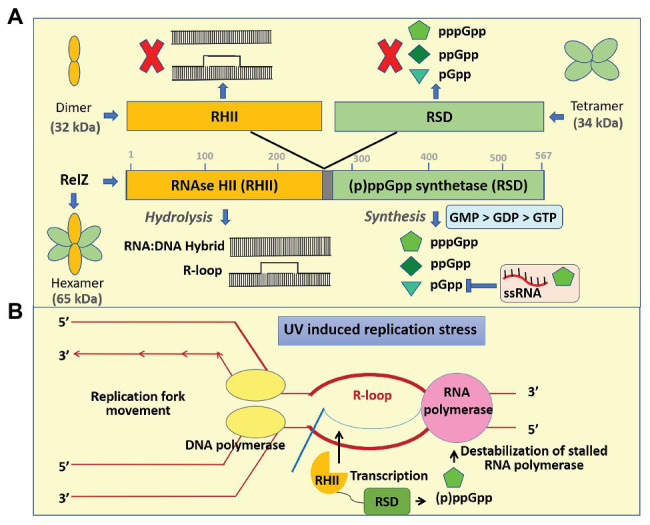
Mechanism of regulation and functions of RelZ. **(A)** RelZ contains an RNAse HII (RHII) domain in tandem with the (p)ppGpp synthetase domain (RSD, RelA SpoT nucleotidyl transferase domain). Full-length protein in *cis* has both RNAse HII activity and (p)ppGpp synthetase activity whereas neither the purified domain variants in isolation nor on *trans* complementation can function independently. RelZ can hydrolyze RNA:DNA hybrid as well as R-loop. It can synthesize pGpp, ppGpp, and pppGpp having the substrate preferences as GMP > GDP > GTP. pGpp synthesis is negatively regulated by ssRNA and high concentrations of pppGpp. From our earlier studies on active site mutational analysis, gel filtration chromatography followed by native PAGE revealed that the N-terminal RHII domain is monomeric and C-terminal RSD domain is tetrameric upon isolation. The full-length active protein is, however, hexamer in solution. We also found that RelZ and all the mutant variants of the full-length RelZ remain as hexameric form in solution. **(B)** In our previous study, we demonstrated that RelZ can hydrolyze R-loops in *Escherichia coli* exposed to UV stress. RelZ gene expression was upregulated under UV stress, and this gene-deleted strain showed increased R-loop accumulation as compared to the wild type. Based on these results, we proposed a model for the physiological function of RelZ. UV stress leads to increased R-loop formation and replication–transcription conflicts. Under UV stress, RelZ expression is upregulated than the conventional RNase HI and HII. Its RHII activity removes the R-loops and the stalled RNA polymerase is destabilized indirectly by (p)ppGpp. Thus, RelZ plays an important role during R-loop-induced replication stress response in *Mycobacterium smegmatis*. **(B)** is adapted and redrawn from Krishnan et al. (2016).

In *M. smegmatis*, MSMEG_5849 codes for a bifunctional protein MS_RHII-RSD (renamed as RelZ), which has a C-terminal RSD domain similar to the other SAS but is different from them due to the presence of N-terminal RNase HII domain in the same polypeptide chain (Figure 3). RelZ efficiently hydrolyze RNA–DNA hybrids (Murdeshwar and Chatterji, 2012) and R-loops (Krishnan et al., 2016). R-loops have a major role in replication–transcription conflicts and lead to stalled arrays of RNA polymerase to block the replication fork movement, thereby promoting replication stress (Drolet, 2006; Poveda et al., 2010; Stirling et al., 2012). This stress can be efficiently managed by two mechanisms: R-loop removal by RNase HII (Aguilera and García-Muse, 2012) and destabilization of stalled RNA polymerase by (p)ppGpp synthesis (Cashel et al., 1996; Ross et al., 2013). RelZ possesses both these important activities (RNase HII and (p)ppGpp synthetase) in a single polypeptide. Our previous study (Krishnan et al., 2016) showed that under UV stress, RelZ removes the accumulated R-loops in RNase H-deficient *E. coli*, and *relZ* expression is upregulated in *M. smegmatis* to remove the R-loops generated due to UV stress. Based on these results, we proposed a model to explain the function of RelZ. Upon UV stress, the levels of RelZ increase within the cell. Any R-loops formed are removed by the RNase HII and (p)ppGpp helps to destabilize the stalled RNA polymerase *via* an unknown mechanism to rescue the cells from replication stress (Krishnan et al., 2016). In addition, RelZ mediates antibiotic tolerance in *M. smegmatis* but does not impact biofilm formation significantly (Petchiappan et al., 2020).

Active site mutational studies of RelZ revealed that inactivation of one domain does not affect the activity of the other domain. However, the purified subdomains are nonfunctional when separated and expressed independently (Figure 3). This kind of domain interdependence was extensively characterized, and the results showed that the full-length RelZ is essential for its function and it is a hexamer (Krishnan et al., 2016). The synthetic subdomain of RelZ is a tetramer in solution like the other solved structures of RelP and RelQ (Steinchen et al., 2015, 2018, 2020; Steinchen and Bange, 2016). Petchiappan et al. (2020) showed that RelZ prefers guanosine monophosphate (GMP) as a substrate and synthesizes pGpp. To understand the difference between pGpp and ppGp, the reaction mixture was treated with NaOH that hydrolyzes only pGpp. From thin layer chromatography, it was shown that Rel hydrolyzes pGpp to GMP and pyrophosphate as evidenced by the comigration of the radiolabeled product with the purified pyrophosphate whereas RelZ showed weak hydrolysis. We found that ssRNA inhibits RelZ-mediated pGpp synthesis, but R-loop did not show any effect (Petchiappan et al., 2020). The pGpp synthesis activity of the RelZ is inhibited by pppGpp whereas ppGpp and pGpp did not have significant effect. Therefore, we infer that the cellular pppGpp levels determine the RelZ-mediated synthesis, whereas ssRNA and pppGpp carefully regulate it. The altered cell surface properties of Δ*relZ* strain indicated that RelZ plays a role in cell wall metabolism (Petchiappan et al., 2020).

*Mycobacterium tuberculosis* has a SAS encoding (p)ppGpp synthetase, Rv1366. But it has no RNase H domain and it is incapable of synthesizing (p)ppGpp (Nanamiya et al., 2008; Weiss and Stallings, 2013; Bag et al., 2014). Few RHII-RSD dual-domain orthologs were identified from Mycobacteria; *Mycobacterium vanbaalenii* (YP_995923.1), *Mycobacterium tusciae* (ZP_09680741.1), and *Mycobacterium gilvum* (YP_001132882.1). However, RelZ is the only dual-domain mycobacterial SAS characterized so far. RelZ type of SAS with RNase H and (p)ppGpp synthetase domains are found only in the environmental species and they are absent in the pathogenic species of mycobacteria.

## SAS Synthesize pGpp

Recently, SAS proteins but not Rel are found to use GMP as a substrate and synthesize pGpp, a third alarmone which makes the alarmone group representation from (p)ppGpp to (pp)pGpp (Gaca et al., 2015b). pGpp can function like (p)ppGpp as well and may have different functions which is not regulated by (p)ppGpp (Gaca et al., 2015a). The pGpp can be hydrolyzed by Rel, like the hydrolysis of (p)ppGpp (Gaca et al., 2015b; Yang et al., 2019). In *B. subtilis*, RelP and RelQ are shown to synthesize ppGp or pGpp. (Tagami et al., 2012). RelQ from *E. faecalis* is an efficient producer of pGpp (Gaca et al., 2015a). RelQ and RelP of *S. mutans* showed much weaker pGpp synthesis activity upon comparison with RelQ_Ef_. RelP and RelQ of *S. aureus* and RelS_Cg_ of *C. glutamicum* synthesize pGpp along with (p)ppGpp. ppGpp/pGpp effectively reduce the intracellular levels of GTP and these guanine nucleotides are synthesized only when RelA is inactive in the cells (Ruwe et al., 2017). The synthesis of pGpp will become relevant only when the GMP levels in the cells are increased like GTP level. Such kind of GMP accumulation has been reported in *B. subtilis* (Liu et al., 2015). It was also speculated that pGpp may be involved in stretching the stress response after the depletion of GTP and GDP in the cell (Gaca et al., 2015b; Ruwe et al., 2017). However, pGpp regulates the purine synthesis but does not involve in ribosome biogenesis (Tagami et al., 2012; Yang et al., 2020).

## SAS Synthesize (p)ppApp

Recent studies by Ahmad et al. (2019) and Jimmy et al. (2020) revealed that SAS not only synthesize ppGpp but also synthesize ppApp. In *P. aeruginosa*, a secreted toxic effector of T6SS was identified as Tas1. Though the crystal structure of Tas1 is similar to the other (p)ppGpp synthetases, it does not synthesize (p)ppGpp but produces (p)ppApp (Ahmad et al., 2019). Another SAS that produces (p)ppApp was identified in *C. marina* FaRel. The toxicity of this toxSAS is mediated by ppGpp and ppApp followed by the depletion of intracellular GTP and ATP pools (Jimmy et al., 2020).

## SAS Bind to ssRNA

Hauryliuk and Atkinson (2017) reviewed the RNA-binding properties of SAS. Beljantseva et al. (2017) discovered that RelQ_Ef_ activity is inhibited when it binds to ssRNA. RNA binds to RelQ in a sequence-specific manner with GGNGG, a putative Shine–Dalgarno-like consensus sequence. pppGpp strongly counteracts the inhibition by RNA and destabilizes the RNA:RelQ complex. In this way, RelQ has both enzyme activity and RNA-binding property. In a RelQ:RNA complex, (p)ppGpp synthesis and pppGpp binding are mutually incompatible. Hence, there is a possibility that the RelQ:RNA complex acts a regulatory switch between inactive and active forms of the enzyme. ssRNA and pppGpp compete with each other to bind into the central cleft of the homotetramer, but this property is not conserved in RelP of *S. aureus*, because pppGpp is not an allosteric regulator of RelP. The central cleft in the RelP tetramer could be an allosteric site bound by other small molecules (Manav et al., 2018; Steinchen et al., 2018).

The RNA-binding property of RelQ can be compared with that of RelZ since the ssRNA inhibits the activity of RelZ (Petchiappan et al., 2020). Since RelZ is involved in R-loop-mediated replication stress (Krishnan et al., 2016), (p)ppGpp synthesis can occur by sensing the R-loops. Once the RNase H cleaves the R-loop into dsDNA and ssRNA (Dutta et al., 2011), the replication stress is relieved and hence the (p)ppGpp synthesis stops. This could be the reason for ssRNA showing inhibitory effect on RelZ-mediated alarmone synthesis. Structural analysis of RelZ is in progress to understand the RelZ:ssRNA complex.

Arresting the (p)ppGpp synthetase activity using (p)ppGpp analogues is emerging as a clinically important method in eradicating persistent infections (de la Fuente-Núñez et al., 2014; Andresen et al., 2016; Petchiappan and Chatterji, 2017; Syal et al., 2017; Dutta et al., 2019). Similarly, the ssRNA-binding property of the SAS can be explored to regulate the SAS-mediated (p)ppGpp synthesis. Mutant huntingtin protein that causes Huntington’s disease was selectively and effectively inhibited by ss siRNA approach (Yu et al., 2012). According to Lima et al. (2012), the identification of potent ssRNA would provide an easy route to therapeutics than dsRNA. ssRNA do not require special formulations for tissue penetration (Bennett and Swayze, 2010), whereas the ds siRNAs need to undergo complex and expensive lipid formulations (Tao et al., 2011). Nucleic acids not only recognize specific target sequences by complementary base pairing but they can interact with proteins and this property is currently being explored in therapeutics (Roberts et al., 2020).

## Conclusion

The co-evolution of SAS along with Rel, redundant (p)ppGpp synthetases, and multiple types of closely related alarmones in bacteria is intriguing. (p)ppGpp is a key factor for biofilm formation, antibiotic tolerance, virulence, and persistence in many pathogenic bacteria. Therefore, inhibition of (p)ppGpp synthesis will inhibit the long-term survival of the pathogen. Therefore, finding an inhibitor to prevent (p)ppGpp synthesis is of high therapeutic interest. In addition to that, ssRNA with specific binding sequence could be a supplementary therapeutic element to inhibit the SAS-dependent (p)ppGpp synthesis because SAS is an RNA-binding protein. The discovery of SAS has not only augmented the prospects of stringent response but also adds value to the upcoming field of RNA therapies.

## Author Contributions

DC and SK conceptualized and wrote the manuscript. All authors contributed to the article and approved the submitted version.

### Conflict of Interest

The authors declare that the research was conducted in the absence of any commercial or financial relationships that could be construed as a potential conflict of interest.
